# Engagement and partnership with consumers and communities in the co-design and conduct of Research: Lessons from the INtravenous iron polymaltose for First Nations Australian patients with high FERRitin levels on haemodialysis (INFERR) clinical trial

**DOI:** 10.1186/s40900-024-00608-9

**Published:** 2024-07-15

**Authors:** Stephanie Long, Cheryl Ross, Joan Koops, Katherine Coulthard, Jane Nelson, Archana Khadka Shapkota, Leiana Hewett, Jaclyn Tate-Baker, Jessica Graham, Rose Mukula, Cynthia Tetteh, Libby Hoppo, Sajiv Cherian, Basant Pawar, Heidi Lee Chmielewski, Lorna Murakami Gold, Geetha Rathnayake, Bianca Heron, Teana Brewster-O’Brien, Vijay Karepalli, Louise Maple-Brown, Robert Batey, Peter Morris, Jane Davies, David Kiran Fernandes, Sajan Thomas, Asanga Abeyaratne, Paul D. Lawton, Federica Barzi, Sean Taylor, Mark Mayo, Alan Cass, Sandawana William Majoni

**Affiliations:** 1 Division of Wellbeing and Preventable Chronic Diseases, Menzies School of Health Research, Charles Darwin University, Northern Territory, Australia; 2 Top End INFERR Clinical Trial Indigenous Reference Group (The Top End Medical Iron Research and Study Advisory Group), Menzies School of Health Research, Northern Territory, Australia; 3 Central Australia INFERR Clinical Trial Indigenous Reference Group (The First Nations Iron Study Advisory Group - Central & Barkly), Menzies School of Health Research, Central Australia, Northern Territory, Australia; 4 Department of Nephrology, Division of Medicine, Royal Darwin Hospital, NT Health, P.O. Box 41326, Northern Territory, Casuarina, Darwin Australia; 5 Department of Endocrinology, Division of Medicine, Royal Darwin Hospital, NT health, Northern Territory, Darwin Australia; 6 Department of Infectious Diseases, Division of Medicine, Royal Darwin Hospital, Northern Territory, Darwin Australia; 7 Northern Territory Medical Program, Flinders University, Royal Darwin Hospital Campus, Northern Territory, Darwin Australia; 8 Department of Nephrology, Division of Medicine, Alice Springs Hospital, NT health, Alice Springs, Northern Territory, Australia; 9 Chemical Pathology–Territory Pathology, Department of Health, Northern Territory Government, Northern Territory, Australia; 10 Child Health Division, Menzies School of Health Research, Charles Darwin University, Northern Territory, Australia; 11 Department of Pediatrics, Division of Women, Children and Youth, Royal Darwin Hospital, NT health, Northern Territory, Darwin Australia; 12 The Central Clinical School, Monash University & Alfred Health, Melbourne, Australia; 13 UQ Poche Centre for Indigenous Health, The University of Queensland, St Lucia Queensland, 4067 Australia; 14 Global and Tropical Health Division, Menzies School of Health Research, Charles Darwin University, Northern Territory, Australia; 15 Northern Territory Department of Health, NT health, Northern Territory, Australia; 16 Flinders University and Northern Territory Medical Program, Alice Springs Campus, Alice Springs, Northern Territory, Australia; 17 Flinders University Centre for Remote Health, Alice Springs, Northern Territory, Australia

**Keywords:** Engagement, Partnership, Consumers, Communities, Aboriginal and/or torres strait Islander peoples, First nations peoples, Research, Reference groups, INFERR, Clinical trial, Haemodialysis

## Abstract

**Background:**

Engagement and partnership with consumers and communities throughout research processes produces high quality research meeting community needs and promoting translation of research into improved policy and practice. Partnership is critical in research involving Aboriginal and/or Torres Strait Islander people (First Nations Peoples) to ensure cultural safety. We present lessons from the design, implementation and progress of the National Health and Medical Research Council funded INtravenous iron polymaltose for First Nations Australian patients with high FERRitin levels on hemodialysis (INFERR) clinical trial.

**Main body:**

The trial was designed to understand the benefits and harms of iron therapy in First Nations Australians on haemodialysis with anaemia and hyperferritinaemia. The lack of evidence for treatment was discussed with patients who were potential participants. A key element ensuring safe conduct of the INFERR trial was the establishment of the Indigenous Reference Groups (IRGs) comprising of dialysis patients based in the Top End of Australia and Central Australia. Two IRGs were needed based on advice from First Nations communities and researchers/academics on the project regarding local cultural differences and approaches to trial conduct. The IRGs underpin culturally safe trial conduct by providing input into study materials and translating study findings into effective messages and policies for First Nations dialysis patients. Throughout the trial conduct, the IRGs’ role has developed to provide key mechanisms for advice and guidance regarding research conduct both in this study and more broadly. Support provided to the IRGs by trial First Nations Research Officers and independent First Nations researchers/academics who simplify research concepts is critical. The IRGs have developed feedback documents and processes to participants, stakeholders, and the renal units. They guarantee culturally safe advice for embedding findings from the trial into clinical practice guidelines ensuring evidence-based approaches in managing anaemia in haemodialysis patients with hyperferritinaemia.

**Conclusion:**

Active consumer and community partnership is critical in research conduct to ensure research impact. Strong partnership with consumers in the INFERR clinical trial has demonstrated that First Nations Consumers will engage in research they understand, that addresses health priorities for them and where they feel respected, listened to, and empowered to achieve change.

**Supplementary Information:**

The online version contains supplementary material available at 10.1186/s40900-024-00608-9.

## Abstract (plain language summary)

In this paper, we present the importance of actively involving consumers in the planning, implementation and conduct of research using the example of a clinical trial among Aboriginal and/or Torres Strait Australians (First Nations Australians) who have kidney disease and are currently receiving haemodialysis. The study assesses how safe and effective it is for people on dialysis to receive iron given through the vein during dialysis when they have anaemia and high levels of a blood test called ferritin, a test used routinely to measure iron levels. Two consumer reference groups of First Nations patients on dialysis, one based in the Top End of Australia and the other based in Central Australia, are supported by First Nations Research Officers and Research Academics to make sure that the research is performed in a way that involves, respects and values First Nations participation, culture, and knowledge. Active consumer and community partnership in this study has supported robust research governance processes which we believe are crucial for knowledge translation to have a positive impact for patients.

**Supplementary Information:**

The online version contains supplementary material available at 10.1186/s40900-024-00608-9.

## Background

There is clear evidence of benefits on health outcomes of embedding research into health care delivery [1–3]. Evidence-based practice is key to high quality patient-centred care. The World Health Organization (WHO) has recognised that high-quality research is essential to achieve the highest possible level of health [4]. Research, and the evidence generated, are critical to improving global health and health equity.

The WHO and United Nations Children’s Fund (UNICEF) Declaration of Alma‑Ata from 1978 states that people have the right to participate individually and collectively in the planning and implementation of their health care [5]. Active engagement and partnership with consumers and community members throughout research conduct produces research that meets the needs of the community and promotes the translation of research into improved policy and practice [6]. Consumer and community involvement should not be tokenistic but represent an active and genuine partnership.

The Australian National Health and Medical Research Council (NHMRC) clearly outlines the benefit of consumer and community involvement in research. Public benefit should include research being conducted that is relevant to community needs; greater public awareness of, and support for, science and research; and more effective translation of research to deliver improved health outcomes [7].

Benefits to researchers and research institutions should include increased community relevance through research priorities and projects being informed by consumer and community perspectives and lived experiences; greater public confidence in research through improved openness and transparency, communities being better informed and having a greater understanding of research; and increased opportunities to continuously improve the quality of research [7]. To maximize the value of research, researchers should seek consumer and community involvement in the early planning stages through building relationships and identifying community priorities for research.

## Consumer and community engagement in research involving first nations peoples

Consumer and community engagement is critical in Aboriginal and/or Torres Strait Islander peoples (First Nations Peoples) (see Table 1 for definitions) research as this empowers them to have a say in how research with First Nations communities is conducted [8–11]. (see Table 1 for definitions). First Nations Peoples should partner in determining research priorities that address key health and wellbeing needs of individuals and communities and to meaningfully contribute to how the research is conducted and translated [12]. Consumer and community involvement is critical to ensure that research is conducted in a way that is culturally safe (see Table 1 for definition). Collaborating with First Nations consumer or community reference groups fosters productive relationships and trust within the partnership, helps to validate research findings and enables the translation of research into practice.

**Table 1:** Glossary of definitions and terms used in the manuscript

Aboriginal and or Torress Strait Islander, First Nations, Indigenous	In this manuscript, “First Nations” encompasses Aboriginal and/or Torres Strait Islander peoples, the First Nations people of Australia. In the Northern Territory, the proportion of people who identify as Torres Strait Islander is very small and the term Aboriginal is widely used to refer to First Nations peoples. Although First Nations peoples is preferred to refer to Aboriginal or Torres Strait Islander or Indigenous peoples, the INFERR clinical trial Indigenous Reference groups (IRGs) have recommended their preferences on the use of the terms as presented in the manuscript. The two IRGs were happy with the general use of the term Indigenous Reference Groups. So, the terms “First Nations”, “Aboriginal” and “Indigenous” were used depending on the context although we have consistently and mostly used the term “First nations” in most cases throughout the manuscript as advised by the IRGs.
Consumer	Refers to First Nations dialysis patients who use (or may use) the health services in the NT, or someone who provides support for First Nations dialysis patients using the health services. Consumers can be patients, carers, family members or other support people. In other sections, for example in the Background section of the manuscript, Consumer refers to people who have lived experience of a health issue and use a health service. They might receive health care or advice, or otherwise use health care services. They include patients, their friends, families, carers and members of the general public. In the INFERR clinical trial, Consumers refer to Aboriginal or First Nations patients on dialysis.
Community	Is a group of people with diverse characteristics who are linked by social ties, share common perspectives, and engage in joint action in geographical locations or settings. In the INFERR clinical trial, community refers to Aboriginal (First Nations) renal community or the Top End or Central Australian Aboriginal Community or renal community
Culturally safe or cultural safety	Cultural safety is about overcoming the power imbalances of places, people and policies that occur between the majority non-Indigenous position and the minority Aboriginal and Torres Strait Islander person so that there is no assault, challenge, or denial of the Aboriginal and Torres Strait Islander person’s identity, of who they are and what they need. Cultural safety is met through actions from the majority position which recognise, respect, and nurture the unique cultural identity of Aboriginal and Torres Strait Islander people. Only the Aboriginal and Torres Strait Islander person who is recipient of a service or interaction can determine whether it is culturally safe. It requires practitioners to deliver safe, accessible, and responsive health care that is free of racism by: recognising and responding to the power imbalance between practitioner and patient reflecting on their knowledge, skills, attitudes, practicing behaviours, and conscious and unconscious biases Culturally safe examples within the INFERR trial context: we are led by the patients’ Aboriginal cultural beliefs and practices specific to their cultural/ language group - this guidance is provided to us by the IRG members who are representatives of their renal units and patients within those units. Examples of culturally safe practices within the INFERR trial: 1) In our study we have applied the IRGs’ demanded culturally safe practices within the consent process such as ensuring a safe space by speaking one on one with patients regarding their health matters and not in an environment where other patients/ people can hear, ensuring interpretation of key messages is predominantly done in their language and with pictures in addition to written text. 2) Patients always prefer oral consent / information processes as Indigenous languages and history are passed on orally not in written form. 3) We have a male nurse to approach and consent male participants in central Australia as required by the IRGs. 4) Giving time for patients to pause and think in silence and not interrupt their thought process is essential. 5) Having an Aboriginal (First Nations) research officer consent participants with the research nurse to offer cultural support and guidance. 6) Utilizing Indigenous (First Nations) artworks and patients’ stories to tell important information within the INFERR trial that is relatable and relevant to Indigenous (First Nations) renal patients’ lives.
Co-designs	Involves collaboration between researchers and consumers from the onset, in question framing, research design and delivery, and influencing strategy, with implementation and broader dissemination strategies part of its design from gestation. It is a human-centered design methodology used in research-action projects to design a product or service. In the co-design approach, end users (or potential users), that is consumers, or the communities participate in knowledge creation and idea generation alongside researchers and designers. In the INFERR clinical trial, we have involved patients form the start of the trial.
Erythropoietin stimulating agents	Erythropoietin stimulating agents (ESAs) are recombinant versions of erythropoietins (EPO) produced pharmacologically. This term refers mainly to erythropoietins. For example, epoetin, darbepoetin, and methoxy polyethylene glycol-epoetin beta. In the Northern Territory renal units, we predominantly we predominantly use darbepoetin, and methoxy polyethylene glycol-epoetin beta
Groups	This term mainly refers to either the Top End or Central Australia or both Indigenous (First Nations) Reference Groups in the INFERR clinical trial. They are also variably referred to as Top End advisory Group and or Central Advisory Group although we have uniformly used the terms Indigenous Reference Group (s) (IRG(s) throughout the manuscript the manasuc

In the Northern Territory (NT), most people requiring kidney replacement treatment identify as First Nations Australians. It is imperative that research teams partner with First Nations consumers and communities so they have a central role in informing, co-designing, and undertaking research in kidney disease. Just like good clinical practice, high quality research should involve consumers and the community in each stage of the research process.

In this paper, we review and outline the lessons learnt from the design and conduct of the NHMRC funded **IN**travenous iron polymaltose for First Nations Australian patients with high **FERR**itin levels on haemodialysis (INFERR) clinical trial [13].

## Conception of the INFERR clinical trial

The presence of hyperferritinaemia is a well-known challenge to the diagnosis and management of iron deficiency anaemia in the NT [14–16]. The effectiveness of erythropoietin stimulating agents (ESAs) or erythropoietins (EPO), which are the main stay of managing anaemia of chronic kidney disease (CKD), is largely dependent on adequate body iron stores. The iron stores are determined by the levels of serum ferritin concentration and transferrin saturation. These two surrogate markers of iron stores are used to guide iron replacement therapy. Most First Nations Australians from the NT with end-stage kidney disease (ESKD) have ferritin levels higher than current guideline recommendations for iron therapy. There is no clear evidence to guide safe and effective treatment with iron in these patients.

In 2009–2010, as part of quality assurance in the Top End Renal Service (TERS), we observed that high doses of EPO and adjuvant iron therapy were used in our maintenance haemodialysis (MHD) patients. We performed two studies seeking to determine the significance of the high serum ferritin observed in First Nations Australian patients on MHD across the NT [14, 15]. Key findings included: ferritin and transferrin saturation (TSAT) may be inadequate for measuring iron stores and should be used with other markers of iron stores, high ferritin could not be fully explained by inflammation, and further studies are needed regarding how best to measure and treat iron deficiency [14, 15] .

Over recent years, renal staff and dialysis patients began to discuss the treatment of anaemia. Some patients asked why they needed to receive iron on dialysis when they have been told EPO was the treatment for anaemia; what effect anaemia could have on their bodies; what would happen if they did not receive iron; what do the iron blood tests mean; and how much iron could be safely given.

Building on the lack of evidence for the effective treatment of anaemia in First Nations patients with hyperferritaemia and discussion within the renal units in the NT, we proposed to conduct a clinical trial to provide evidence to guide care. The INFERR clinical trial was designed to address the uncertainty regarding the safety and efficacy of iron therapy in First Nations Australians with CKD with hyperferritinaemia and evidence of iron deficiency. The trial is a randomized, open label, study to compare hospitalization and death between those receiving intravenous iron polymaltose 400 mg once monthly or those receiving no IV iron. The primary outcome will be the differences between the two study arms (Arm A: Receiving intravenous iron and arm B: Receiving no iron unless there is decision by the clinicians to give iron) in the risk of hospitalisation with infection or death [13].

## Involving consumers and community members in the INFERR clinical trial

The INFERR trial was designed to understand the benefits and harms of iron therapy in First Nations Australians on haemodialysis with anaemia and hyperferritaemia [13]. The lack of evidence for safe therapy of anaemia using iron in this population was clearly identified and discussed with patients on haemodialysis whose health outcomes were directly affected and who supported the development of the INFERR clinical trial [13, 16]. A key element of ensuring the safe conduct of the INFERR trial was the establishment of the INFERR Indigenous Reference Groups (IRGs) comprising of dialysis patients, one IRG based in the Top End of Australia and one IRG in Central Australia. The need for two IRGs was based on advice from First Nations communities and First Nations researchers/academics on the project regarding cultural differences and approaches to trial conduct which needed to be addressed locally.

A key engagement strategy for the INFERR trial was providing each IRG with the support of a First Nations Research Officer employed on the project and an independent First Nations researcher/academic to assist in explaining research concepts as needed.

### Roles and terms of reference (TOR) for IRGs

#### Purpose

The purpose of the IRGs is to provide high level advice and advocacy for the INFERR study participants, dialysis patients and communities engaged in the trial.

#### The IRGs function

**Fig. 1:**
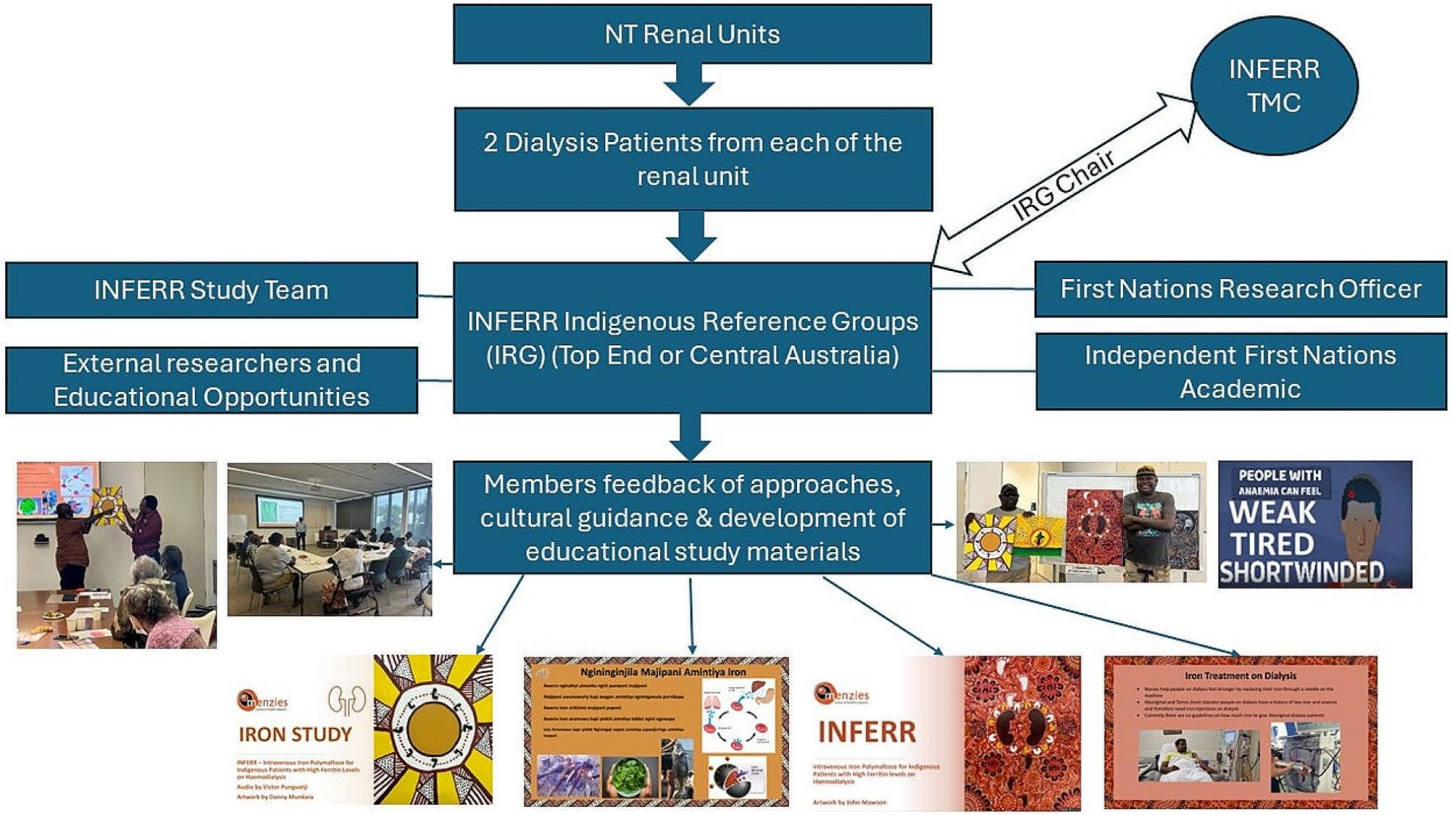
The roles, function, relationship of and feedback from the INFERR clinical trial Indigenous Reference Groups (IRGs)

to provide advice and advocacy on First Nations social, cultural and health issues relevant to research conduct. Key roles include: to facilitate and contribute to the development of health literacy, participant information and consent materials; to advise regarding the conduct and impact of research in participating communities; to advise and direct how to ensure that the study is conducted in a culturally safe manner; to advise regarding knowledge transfer to First Nations communities in the NT; to advise regarding capacity building opportunities for First Nations peoples engaged in the project and to advise regarding dissemination of the study findings and translation of these findings into clinical practice. (see Fig. 1).

#### Composition

The INFERR trial IRGs consist of 7–15 members, all of whom are First Nations Australian dialysis patients. Community members related to dialysis patients may attend IRG meetings as support persons but have no voting powers. The membership includes at least two representatives from each dialysis unit across the Northern Territory. Ideally, one is male, and one is female. Additional consideration is given to ensure regional language groups are represented and a mix of age groups. To become a member, dialysis patients can self-nominate or be nominated by or from communities and community-controlled services which are partners in this study. The only non-consumer voting representative is an independent First Nations Academic/researcher. The trial provides each IRG with a First Nations Research Officer to organize, attend the meetings, and provide study progress reports and secretarial support. A quorum is met when there is 50% plus 1 of the members present.

#### Educational component of the IRG meeting

Most meetings have included an educational component for IRG members on any questions on the research process, health service or any other health related matters; the topic is based on requests from IRG members. The subject matter experts are contacted by the First Nations Research Officer to present information and learnings for members of the IRG. Examples of requested education providers included renal nutritionist, renal transplant coordinators and Health Living NT cardiac education service provider. Each presentation was well received with members engaged and asking questions. This has enabled the trial to provide some reciprocity for members who have kindly agreed to be a member of the IRG. (see Fig. 1).

#### Chairperson

The IRG chairperson ensures that IRG meetings are conducted in accordance with the agreed terms of reference and focus on priorities identified by IRG members. A representative from each IRG, usually the chair, is a member of the INFERR Trial Management Committee (TMC). The chairperson is the primary contact for information flow between the INFERR TMC, clinical trial manager and the members of the IRGs.

IRG members are encouraged to rotate the chairperson position among members. However, this is at the discretion of the IRGs. (see Fig. 1).

#### Independent first nations researchers/academics

Two First Nations researchers/academics, one each for the Top End and the Central Australian groups, who are not otherwise involved in the conduct of trial, are members of the IRGs. They fulfil a supporting role within the group and seek to enhance the autonomy and authority of IRG members. They have strong research track records, but do not necessarily have to have dialysis, kidney disease or clinical trials expertise. These independent research/academic members were appointed to the IRGs by the Chair of the INFERR TMC, in consultation with the TMC.

The independent researchers/academics also ensure that key study documents are understood by IRG members and that the IRG members can make informed and independent decisions. Study educational materials and other participant facing documents are simplified and translated with the IRGs. The First Nations research officers and First Nations researchers/academics aid in simplifying many of the complex study documents for the IRG members to better understand. The IRGs are then able to interpret them for renal patients in their language through the creation of simplified study materials. The researchers/academics are voting members of the IRGs. (see Fig. 1).

#### First Nations research officers

Two First Nations INFERR clinical trial Research Officers (one in the Top End and one in Central Australia) with established connections in the Northern Territory play key roles in consumer and community engagement. The First Nations Research Officers have a thorough understanding of the research process as they have received training, education and induction from the Menzies orientation program which was provided by the project manager and the chief investigators of the trial. They also receive mandatory good clinical practice (GCP) training. (see Fig. 1).

They work closely with all stakeholders, which include government and non-government health service providers, to determine and implement activities that facilitate communication between consumers, researchers, and health services. They are responsible for operational aspects of this engagement including assisting in the development of culturally safe and appropriate resources. They also facilitate the informed consent process for potential participants. Their role involves co-design processes such as development of all study participant facing documents with IRG patient members; for example, consent forms and participant information sheets. They inform and assist with implementation of the clinical trial, including assistance with stakeholder engagement and approvals, and ensure research activities are conducted in a culturally safe manner.

The Research Officers are responsible for the establishment and ongoing function of each IRG. On behalf of the IRG, the research officer arranges meetings, including working with the IRG Chair to develop the agenda, meeting location, invitation of educational subject matter expert, catering, and research team members updates for each meeting. The research officer writes the minutes and distributes the key findings including any concerns identified at the meetings. They are not voting members of the IRGs but are vital in helping facilitate and support the IRG members.

#### Authority of the IRGs

The INFERR trial IRGs function independently of other INFERR trial committees or teams. The IRG is part of the INFERR governance framework and provides recommendations to the TMC. The IRGs provide expert advice to research staff on critical issues relating to engaging renal patients within the dialysis clinics. This advice has addressed issues such as culturally safe communication and appropriate consenting processes, reviewing in their meetings how the project continues to involve the community and other stakeholders, interpretation and advice on dissemination of emerging findings from this research project for example sharing of information regarding the high prevalence of markers of liver disease. f. Renal patients participate as active members of the IRG and recruitment as a participant in the actual study is not required. A member from each IRG is nominated by the group as a representative (usually the chair) to sit on the Trial Management Committee. Their role on the TMC is to talk to the recommendations made during the IRG meetings. The TMC will consider these recommendations with final approval resting with the TMC. The IRG representative and the First Nations Research Officers provide feedback of TMC meeting at the subsequent IRG meeting. Although their primary role is related to the clinical trial, the IRGs also provide general feedback to renal services across the NT and other research projects.

#### Custodianship of data

The TMC, operating in accordance with the relevant institutional polices of Menzies School of Health Research, are the custodians of the INFERR trial data. The TMC recognizes the important role of the INFERR trial IRGs in data governance and INFERR trial committees recognize the collective benefit, authority, responsibility, and ethics. (CARE) principles of Indigenous data sovereignty. The INFERR trial IRGs continue to provide guidance regarding acceptable and appropriate data usage and interpretation and dissemination of findings. The INFERR study will establish an Editorial Sub-Committee to oversee relevant data governance issues. The IRGs will provide a member to the Editorial Committee.

#### Time commitments

The INFERR trial IRGs meet at least three times per year and whenever the need arises. The meetings are a mix of face-to-face meetings in Darwin or Alice Springs and teleconference/videoconference for those unable to travel. Face-to-face meetings constitute a half day, including allocated time for education. As required, additional teleconferences or other face-to-face meetings can be called.

As well as extending an invitation to individuals to participate in the INFERR IRGs, upon acceptance, letters are written to members’ employers and/or representative organisations to seek their support and acknowledge their contribution to the project through the release of the INFERR trial IRG member for reference group activities and meetings.

#### Costs

Travel, meals and accommodation costs are covered by the INFERR study. Bookings are made and travel arranged by the First Nations Research Officer.

#### Payments

Where an IRG member is employed, support is sought for members to be paid by their employer to attend the IRGs meetings as part of their professional development and/or regular employment. In the case of an IRG member being self-employed or unemployed, the IRG members are compensated for their time in attendance at an IRG meeting. All members are paid according to the per Menzies School of Health Research Enterprise Bargaining Agreement (EBA) 2018 for the time they attend meetings. The members are paid A$50.40 per hour for a minimum of 3 h. On the day of the meeting, transport to and from the meeting is provided, as well as morning tea and lunch.

#### Meeting processes

The agenda for the IRG meetings follows a standard format in keeping with the agreed purposes of the IRG (Additional document 1). Each meeting consists of the option to have a closed session with voting members and the First Nations Research Officer present.

Members are asked at each meeting if they consent to the recording of the meeting. All recordings are kept and stored by the First Nations Research Officer at Menzies School of Health Research (Darwin) to assist with drafting minutes. Once the minutes are circulated to the group and accepted, the recordings are deleted. All recordings are private and confidential and only accessed by the IRG Chair and the secretarial and administrative support. The minutes including any action items are circulated within one week of the meeting. The agenda and any required reading materials are provided to the IRG at least two weeks prior to each meeting. A brief project progress report is tabled at each meeting by the Clinical Trial Manager or Principal Investigator. Additional meetings of the IRG or meetings with a TMC chair or delegate can be requested at any time. There is continuation of engagement with members between meetings via newsletters and face-to-face catch ups at the renal clinics.

#### Review of IRG TOR or membership

The IRG can review and amend the TOR and the membership of the IRG as needed. Any amendments or modifications to IRG TOR or membership will need to be communicated through the First Nations Research Officers and Clinical Trial Manager to the TMC Chair as soon as possible and be tabled and ratified at the next TMC meeting. (Additional document 2)

Figure 1 provides an outline of the roles including some examples of the feedback provided by the IRGs and the relationship of the IRGs, First Nations research officer, First nations academics and other teams and committees within the INFERR clinical trial.

## Important work of the IRGs in the progress of the INFERR clinical trial

**Fig. 2:**
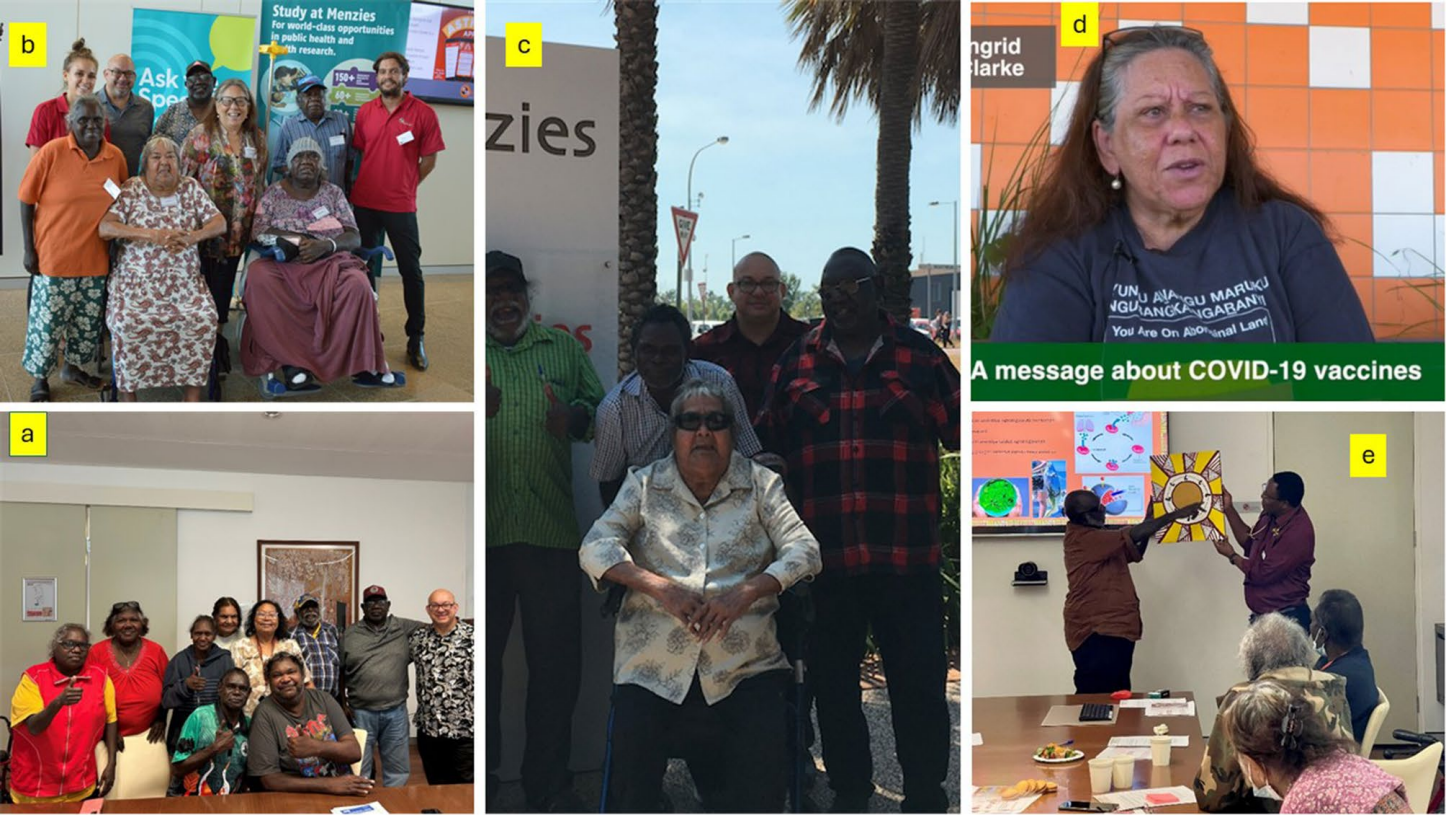
Top End Indigenous Reference Group (IRG) (The Top End Medical Iron Research and Study Advisory Group) (**a**) One of the latest Top End INFERR IRG meeting from left to right: Helen Stevenson, Anne-Marie Puruntatameri (R.I.P), Pauline Kerinauia, Joan Koops (Current Chair), Margret Vigona, Kevin Rogers, Danny Munkara, Mark Mayo (First Nations Academic), Victor Punguatji, Wayne Alum (**b**) First Top End INFERR IRG meeting February 2021: Left to Right: (Top) Stephanie Long (First Nations Research Officer), Mark Mayo (First Nations Academic), Danny Munkara, Ingrid Clarke (former chair) (R.I.P), Kevin Rogers, Anthony Long (First Nations Research Officer) (Bottom) Cathie Nickels (R.I.P) Barbara Patterson (R.I.P) (**c**) Another of Top End IRG meeting: Left to Right: (Top) Helen Stevenson, Kevin Rogers, Victor Punguatji, Mark Mayo, Danny Munkara, Joan Koops (Current Chair of the IRG). (Bottom) Cathie Nickels (R.I.P) **d**) Top End INFERR IRG; A snippet from a covid-19 Menzies video project that the former IRG chair Ingrid Clarke (R.I.P) was able to speak on due to her involvement with the INFERR IRG **e**) Top End INFERR IRG meeting July 2023: Mr. Danny Munkara presenting his Tiwi artwork to be used on the INFERR iron Tiwi booklet. This is an information booklet explaining the role of iron in anaemia and the INFERR clinical trial in one of the Aboriginal Languages, the Tiwi language. **Warning: Aboriginal and Torres Strait Islander readers are advised that this article contains the name and images of Indigenous person who has died used with the permission of their families**

**Fig. 3:**
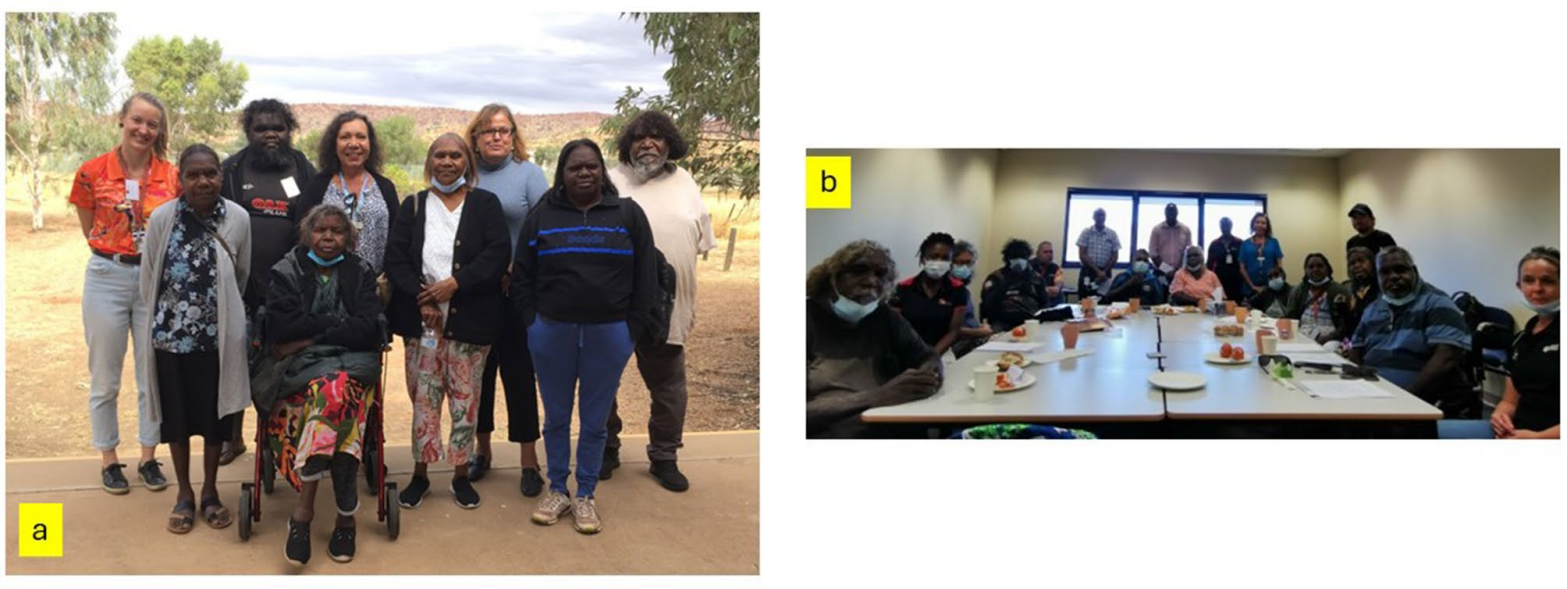
Central Australian Indigenous reference group (IRG) (The Central and Barkly Indigenous Reference Group) (**a**) Central Australia (Central and Barkly) IRG meeting: May 2022 (**b**) Central Australia (Central and Barkly) IRG meeting: March 2023. **Warning: Aboriginal and Torres Strait Islander readers are advised that this article contains the name and images of Indigenous person who has died used with the permission of their families**.

The first participant was recruited in December 2020 in the Top End and July 2021 in Central Australia. Despite extended pandemic-related lockdown periods, during which recruitment for clinical research was halted, as of June 2024, over 440 participants have been recruited out of a target of 576 participants. The IRGs have been meeting in the Top End and in Central Australia since February 2021 and August 2021 respectively and will continue to meet throughout the conduct of the research (Figures 1, 2 and 3). The INFERR IRGs have met 8 times in the Top End and 6 times in Central Australia with a quorum at each of the IRG meetings except for 2 early group building meetings and one meeting which was impacted by an unseasonal, cold and wet weather event in Central Australia (See additional document 2).

The IRG meetings have provided key decisions and feedback for the conduct of the trial. Initial IRG meetings focused on optimal ways that groups could work together, their group name, and the review and development of participant facing documents including the participant information sheets and consent forms. Through this process the IRG recommended and helped to develop educational animated messages in Aboriginal languages articulating the role of iron in the body and using appropriate feedback formats for participants and renal patients. Over time the IRG governance role has developed to include advocacy on renal issues and advice or endorsement for other potential kidney disease research projects.

One of the pivotal roles of the IRGs is the translation of study findings into appropriate health messages and support for translation into effective health policy for First Nations patients on dialysis. New local clinical practice guidelines will be developed to ensure an evidence-based approach to the management of anaemia in haemodialysis patients with high ferritin. For example, the IRGs have already been actively participating in discussions leading to the development of clinical care pathways for dialysis patients with liver disease.

### Key decisions and feedback from the meetings

#### Process for naming the IRGs

In the Top End, the IRG deliberated over their name. With the assistance of the First Nations Research Officer and Academic, the Top End IRG chose the title of *“The Top End Medical Iron Research and Study Advisory Group”* as it was a broad title that covered the research and also the advisory concept of the IRG. The Top End IRG also sought to keep the terms *“Aboriginal”*,* “Indigenous”* and/or *“First Nations”* out of their group’s name as they saw it important to be seen as an advisory IRG not characterised by ethnicity as they saw medicinal iron as the key concept of the study and thus their group. Whilst believing their culture and Indigenous knowledge applied in relation to all their views and advice, the members decided that they did not need the terms *“Aboriginal”*,* “Indigenous”* and/or *“First Nations”* in the name of the group as it is their lived experience of health and wellbeing that remains at their centre and thus the IRG’s centre in its approach (Fig. 2).

In central Australia, the IRG discussed the Top End IRG process and decision regarding a name. After general discussion, it was decided to keep *“Indigenous”* or *“Aboriginal”* or *“First Nations”* in the title, but to use a common or shared terminology. The Research Officer advised that this might be challenging as the IRG is made up of different language groups with their own ways of referring to or naming community members. The IRG agreed to deliberate overtime with the Research Officer following up with each member. The group settled on the name *“First Nations Iron Study Advisory Group - Central & Barkly”* (Fig. 3).

#### Reviewing of and recommending amendments to all participants facing documents and clinical trial procedures

The IRGs revised all participant facing documents such as participant information sheets (PIS) and consent forms and simplified them for potential participants. For example, they have recommended changes to participant information sheets and transferring the information to flip charts, using pictorial presentations and translating the information into Aboriginal languages. Amendments were sent to the ethics committee, including the Aboriginal ethics subcommittee and were all approved.

#### Reviewing and production of study information

Documents, visual aids and videos explaining anaemia, iron deficiency and their management in general and the INFERR clinical trial were produced by the IRGs coordinated by the First Nations Research Officers.

#### Providing feedback on the cultural safety and appropriateness of the conduct and progress of the INFERR trial

Each IRG meeting has provided feedback to improve the conduct of the trial to remain culturally safe and appropriate. This role has grown to enable the IRGs to provide feedback and guidance regarding a broader range of issues as they impact renal service provision and research co-design and conduct in both Top End and Central Australia. Advice has also been provided to the TMC and INFERR investigators to update participants, renal patients and the community on any important information, interim results or results of other pre-specified analyses in the protocol from the trial.

#### Reviewing and providing feedback to presentations from other projects from Menzies and other NT health researchers

In addition to their role on the INFERR clinical trial, the IRGs have also reviewed and provided feedback to several other research projects affecting First Nations Australians in the NT. Examples of these include the cultural safety of preoperative assessment and preparation for surgery, research being carried out in the intensive care units, COVID and flu vaccination studies and a clinical trial (the SWIFT study) planned for dialysis units across Australia.

#### Provide consumer feedback on appropriate service development of renal services to the health leadership of the NT renal services in the top end and in central Australia

Involving consumers and community members in service development is an important step towards developing and delivering effective, person-centered health care. The IRGs have provided feedback regarding issues affecting them which include: the need for improved culturally safe and appropriate communication from health care staff, being respected when communicating with dialysis nurses and other staff, discomfort of new dialysis chairs, problems with transport of patients to dialysis units and the need to develop renal services to provide treatment for patients closer to their home communities. This feedback has been escalated to the appropriate health service leadership and stakeholders who are now reviewing and implementing change.

#### Translation of the outcomes from the INFERR clinical trial sub studies into clinical practice

Important findings have emerged from two sub studies during the conduct of the INFERR clinical trial. The IRGs have played a key role in reviewing and providing feedback to the INFERR trial investigator team and renal patients on these findings. Results from the cross-sectional comparison of two assay platforms used to measure ferritin levels in the NT showed that ferritin results were consistently 36–44% higher using one platform than the other platform. The bias was up to 49%. These results have led to the adjustment of ferritin levels in standard clinical guidelines and the INFERR trial protocol inclusion criteria [16]. Results of the protocol pre-specified second sub study assessing liver stiffness using FibroScans^®^ have shown a high prevalence of elevated FibroScan^®^ liver stiffness measurements. Liver disease has been identified as a major clinical issue and specific clinics are being set up to manage this in the dialysis units.

## The future

Throughout conduct of the INFERR trial, the role of the IRGs has developed so that they provide a key mechanism for advice and guidance regarding research conduct both in relation to this study and more broadly. We seek funding to further develop consumer engagement materials, so they are accessible and appropriate for First Nations Australians from remote communities across Northern and Central Australia. We will also seek funding to establish a liver clinic in people on dialysis, to address a key interim finding from the INFERR study regarding the high prevalence of comorbid liver and kidney disease. The IRGs will assist with identifying knowledge gaps for educational resources around anaemia management and translation of the results of the clinical trial into practice guidelines.

Building on broader roles the IRGs have assumed, it is clear that the IRGs will play a critical role in feedback regarding renal service delivery and consumer engagement in research for the NT. Translation of consumer materials especially educational resources into First Nations languages has been a constant message from the IRGs and will require significant time and funding going into the future with the INFERR trial and other projects. This is rarely factored into research projects and clinical care.

## Conclusion

What lessons have we learnt? The success of the INFERR trials consumer engagement process was early engagement with First Nations dialysis patients through the key position of our two First Nations Research Officers. This provides an appropriate cultural safeguard for dialysis patients to feel empowered to ask questions and to say no without consequences. The First Nations Academics are essential to guide research understanding and support during the IRG meetings. Embedding the IRG into the governance structure of the trial provided the IRG with the authority to make recommendations and provided a direct pathway for access to the TMC. Feedback to the IRG through the First Nations research officer and the IRG TMC representative, regarding progress in addressing their recommendations has provided validation to IRG members that their input is truly valued by the TMC. Regular meetings that include educational opportunities for members help ensure enthusiasm and attendance. Finally, it is essential to have financial support to run the IRG meetings, to pay members appropriately for their contribution, and provide the support that is required.

In this commentary, we have provided details of how effective engagement of First Nations consumers through Indigenous Reference Groups (IRGs) have been core to the governance of the INFERR clinical trial. Our established IRGs provide First Nations Governance oversight and ensure the trial is conducted in a culturally safe manner in strong partnership with consumers. Consumer and community involvement is key for conducting research that will result in real health improvement [17]. The experience and lessons learnt from the role of the IRGs in the INFERR clinical trial can be scaled to health research in other communities. This is not only limited to Indigenous populations but also relevant to other culturally and linguistically diverse populations. The role of study reference groups is primarily to advise and advocate for appropriate and ethical conduct of a particular research project. However, depending upon need and with the provision of institutional and health service support, the role can expand to include a whole of service feedback and a broader research advisory and advocacy focus.

### Electronic supplementary material

Below is the link to the electronic supplementary material.

Supplementary Material 1

Supplementary Material 2

## Data Availability

No datasets were generated or analysed during the current study.

## Abbreviations

ANZDATA: Australia and New Zealand Dialysis and Transplantation Registry
CAHREC: Central Australia Human Research Committee
DSMB: Data Safety and Monitoring Board
HREC: Human Research Ethics Committees
EBA: Enterprise bargaining agreement
ESA: Erythropoietin stimulating agents
EPO: Erythropoietin
GCP: Good Clinical Practice
INFERR: INtravenous iron polymaltose for First Nations Australian patients with high FERRitin levels on haemodialysis
IRGs: Indigenous Reference Groups
IV: Intravenous
Menzies: Menzies School of Health Research
NHMRC: National Health and Medical Research Council
NT: Northern Territory
RDH: Royal Darwin Hospital
RGO: Research Governance office
TERS: Top End Renal Service
TMC: Trial Management Committee
TOR: Terms of reference
WHO: World Health Organization
