# Supplementary material for: Engagement and partnership with consumers and communities in the co-design and conduct of Research: Lessons from the INtravenous iron polymaltose for First Nations Australian patients with high FERRitin levels on haemodialysis (INFERR) clinical trial
Source: Res Involv Engagem. 2024 Jul 15;10:73. doi: 10.1186/s40900-024-00608-9 (PMC11250943; doi:10.1186/s40900-024-00608-9)
Supplement: Supplementary file 1 — Supplementary Material 1 [file 40900_2024_608_MOESM1_ESM.docx]

**Format of the agenda of IRG meetings**

1. Acknowledgement of Country and Traditional Owners

2. Present / Apologies (quorum)

3. Minutes of the previous meeting

4. Project progress report/ feedback from INFERR TMC meetings (Usually delivered by INFERR trial manager, INFERR trial First Nations Research Officer and the INFERR trial principal investigator or proxy)

5. Matters arising

6. Other business

7. Next meeting

8. Close
