# Supplementary material for: Engagement and partnership with consumers and communities in the co-design and conduct of Research: Lessons from the INtravenous iron polymaltose for First Nations Australian patients with high FERRitin levels on haemodialysis (INFERR) clinical trial
Source: Res Involv Engagem. 2024 Jul 15;10:73. doi: 10.1186/s40900-024-00608-9 (PMC11250943; doi:10.1186/s40900-024-00608-9)
Supplement: Supplementary file 2 — Supplementary Material 2 [file 40900_2024_608_MOESM2_ESM.docx]

**Summary of the INFERR clinical trial Indigenous Reference Groups (IRGs) meetings since the start of the study**

**Top End**

**From the Terms of Reference (TOR)**

**Composition:** The IRG would consist of 7-15, of whom all will be Aboriginal or Torres Strait Islander peoples (First Nations Peoples) on haemodialysis dialysis except the independent First Nations Academic/Researcher. One (1) member will be an independent First Nations academic, with two (2) members from each of the study sites in the Top End (Nightcliff, Palmerston, Katherine and Tiwi Islands). Of the two members from each site, ideally one (1) will be male and one (1) will be female. The members can self-nominate or be nominated by or from Aboriginal communities and community-controlled services which are partners in this study. The INFERR Clinical Trial Manager will attend the meetings, provide study progress reports and secretarial support only.

A quorum would be met when there is 50% plus 1 of the members present: For example, if there are 10 members on the IRG, a quorum will be reached if at least 6 will attend each meeting.

**Table S1: Meeting attendances for the Top End IRG meetings in the course of the INFERR study**

| **Meeting number** | **Meeting Date** | **Number attended excluding Academic** | **Academic** |
| --- | --- | --- | --- |
| 1 | 20/10/2020 | 7 | Plus the academic |
| 2 | 25/02/2021 | 6 | Plus the academic |
| 3 | 18/05/2021 | 8 | Plus the academic |
| 4 | 16/09/2021 | 9 | Plus the academic |
| 5 | 26/04/2022 | 9 | Plus the academic |
| 6 | 09/11/2022 | 8 | Plus the academic |
| 7 | 07/03/2023 | 9 | Plus the academic |
| 8 | 04/07/2023 | 8 | Plus the academic |

The quorum for meetings is 6 to ensure adequate representation of patients can be met for each Top-End renal clinic. There are roughly 12-15 active members in the group and due to dialysis/life schedules and other essential commitments attendances vary from this number hence why the quorum is also 6 to ensure half representation is always present within the group.

**Central Australia**

**From the Terms of Reference (TOR)**

**Composition:** The IRG would consist of 7-10 members (ideally no less than 6 members and up to 15 members), of whom all will be Aboriginal or Torres Strait Islander peoples (First Nations peoples). One (1) member will be an independent First Nations academic, with two (2) members from each of the study sites in Central Australia (Flynn Drive, Gap Road and Tennant Creek Dialysis Units). Of the two members from each site, ideally one (1) will be male and one (1) will be female. The members can self-nominate or be nominated by or from Aboriginal communities and community-controlled services which are partners in this study. The INFERR Clinical Trial Manager will attend the meetings, provide study progress reports and secretarial support only.

A quorum would be met when there is 50% plus 1 of the members present: For example, if there are 10 members on the IRG, at least 6 will need to attend each meeting.

**Table S2: Meeting attendances for the Top End IRG meetings in the course of the INFERR study**

| **Meeting**  **number** | **Meeting Date** | **Number attended excluding Academic** | **Academic** |
| --- | --- | --- | --- |
| 1 | 22/07/2021 | 3 – Building the group | Nil |
| 2 | 02/11/2021 | 3 – Building the group | Plus the academic |
| 3 | 19/05/2022 | 9 | Plus the academic |
| 4 | 01/09/2022 | 5 No quorum (Unseasonal cold, wet weather impacted) | Plus the academic |
| 5 | 09/03/2023 | 11 | Plus the academic |
| 6 | 05/10/2023 | 8 | Nil |
